# A Cancer Gene Selection Algorithm Based on the K-S Test and CFS

**DOI:** 10.1155/2017/1645619

**Published:** 2017-05-08

**Authors:** Qiang Su, Yina Wang, Xiaobing Jiang, Fuxue Chen, Wen-cong Lu

**Affiliations:** ^1^School of Communication & Information Engineering, Shanghai University, Shanghai 2000444, China; ^2^Department of VIP Medical Center, The Third Affiliated Hospital of Sun Yat-sen University, Guangzhou 510630, China; ^3^Department of Neurosurgery, Sun Yat-sen University Cancer Center, State Key Laboratory of Oncology in South China, Collaborative Innovation Center for Cancer Medicine, No. 651, Dongfeng Road E, Guangzhou 510060, China; ^4^College of Life Sciences, Shanghai University, Shanghai 2000444, China; ^5^Department of Chemistry, College of Sciences, Shanghai University, Shanghai 200444, China

## Abstract

**Background:**

To address the challenging problem of selecting distinguished genes from cancer gene expression datasets, this paper presents a gene subset selection algorithm based on the Kolmogorov-Smirnov (K-S) test and correlation-based feature selection (CFS) principles. The algorithm selects distinguished genes first using the K-S test, and then, it uses CFS to select genes from those selected by the K-S test.

**Results:**

We adopted support vector machines (SVM) as the classification tool and used the criteria of accuracy to evaluate the performance of the classifiers on the selected gene subsets. This approach compared the proposed gene subset selection algorithm with the K-S test, CFS, minimum-redundancy maximum-relevancy (mRMR), and ReliefF algorithms. The average experimental results of the aforementioned gene selection algorithms for 5 gene expression datasets demonstrate that, based on accuracy, the performance of the new K-S and CFS-based algorithm is better than those of the K-S test, CFS, mRMR, and ReliefF algorithms.

**Conclusions:**

The experimental results show that the K-S test-CFS gene selection algorithm is a very effective and promising approach compared to the K-S test, CFS, mRMR, and ReliefF algorithms.

## 1. Introduction

Big data analysis technology can mine gene information related to diseases and drugs from massive gene data and provide new ideas for drug development as well as disease diagnosis and treatment. Therefore, big data has positive effects on cancer research. Genetic data analysis includes four steps: gene data acquisition, gene data pretreatment, gene selection, and classification model establishment and evaluation. Of these steps, genetic data acquisition is a biomedical process, and the other steps are data mining processes. This paper focuses on the gene selection step in genetic data analysis by exploring the challenges to gene data analysis and effective strategies and methods for gene selection.

According to its relationship with the classifier, the feature (gene) selection method is divided into the filter method, the wrapper method, and the embedded method. The filter method selects the features that contribute to the classification, which is independent of the learning process, and has a higher efficiency and a stronger generalization ability. The wrapper method selects the corresponding feature subsets according to the classification performance of the feature subsets. Depending on the learning process, the wrapper method has a higher accuracy, but it is prone to overadaptability, a poor generalization performance, and low time efficiency. The combination of the filter method and the wrapper method is a new trend in studies of feature selection.

There is a significant difference in the expression value of a discriminative gene between different genotypes. Thus, a series of filter-based gene selection methods, based on parametric statistics, was developed to detect whether there were significant differences between genotypes and to select a subset of genes with significant differences [1, 2]. However, parametric statistical methods need to assume a Gaussian distribution of the data, and the actual genetic dataset usually does not meet the Gaussian distribution hypothesis. Therefore, a nonparametric statistical method, the Wilcoxon rank sum test, is used in gene selection studies. However, the rank sum test can be used to reveal the location of two sample types (the distributions of the values of the two sample types) only when the sample size is large or the measurement level is low (the sample observations have only a small number of values). When the sample size is very small or has the same rank value as the sample with the same rank, it is not appropriate to use the rank sum test for gene selection.

The Kolmogorov-Smirnov (K-S) test is another nonparametric statistical method used to compare the distribution of two sample types. This method is very sensitive to the difference of the distribution of two sample types. It has been successfully applied in the analysis of ovarian cancer gene data, recognition, and other fields [3]. However, an independent nonparametric test method does not take into account the redundancy of the genes in the selection of genes with discriminatory power.

The correlation-based feature selection (CFS) [4, 5] method can efficiently select subsets of genes that are highly correlated with the class and that have low redundancy. However, due to the high-dimensional characteristics of gene datasets, it is very time-consuming to adopt the CFS method for gene selection directly. Therefore, a gene selection algorithm combining the K-S test and CFS is proposed in this paper. Most of the redundant and noise genes are removed by the K-S test, and the genes with a significant distinguishing ability are retained. Then, CFS is used to evaluate the genes that are highly correlated with the class and have low redundancy. A support vector machine (SVM) [6, 7] is used as the classifier to evaluate the gene subsets generated based on accuracy. Finally, the method is compared with the K-S test, minimum-redundancy maximum-relevancy (mRMR) [8], and classic ReliefF algorithms [9]. The experimental results from five gene datasets show that the K-S test-CFS gene selection method is an effective gene selection algorithm.

## 2. Materials and Methods

### 2.1. Datasets Description

In this paper, five classical cancer gene datasets are used: breast cancer [10], lung cancer [11], colon tumor [12], ovarian cancer [13], and leukemia [2]. Detailed information on the datasets is listed in Table 1. To eliminate the influence of different dimensions on the experimental results, the five datasets were *Z*-score standardized as part of the preprocessing.

### 2.2. K-S Test

In this paper, the K-S test was used to determine significant differences between the genes of the tumor patients and those of normal controls. Let *X*_1_, *X*_2_, *X*_3_,…, *X*_*N*_ be a gene *X* from the gene dataset, and the observed value is *x*_1_, *x*_2_,…, *x*_*n*_, where *n* is the sample number of the gene dataset. According to the gene order value, the order of the observations is *x*_(1)_ ≪ *x*_(2)_ ≪ ⋯≪*x*_(*n*)_, and the cumulative distribution function of the gene *X* is defined as follows:

(1) $$\begin{array}{c} F\left(\;x\;\right)=\left\{\begin{array}{cc} 0, & x<x_{\left(1\right)}\\ \frac{k}{n}, & x_{\left(k\right)}\leq x\leq x_{\left(k+1\right)},\;\;k=1,\ldots ,n-1\\ 1, & x>X_{\left(n\right)}. \end{array}\right. \end{array}$$

Assuming that the cumulative distribution functions of the gene to be tested in the tumor sample and the normal sample are *F*_1_(*x*) and *F*_2_(*x*), where the number of observations is the number of positive and negative samples, the K-S test statistic is

(2) $$\begin{array}{c} D=\underset{x}{\max}\left|\;F_{1}\left(\;x\;\right)-F_{2}\left(\;x\;\right)\;\right|. \end{array}$$

According to the K-S test theory, when *D* < *D*_crit_ (the critical value of *D*_crit_ for the level of significance *α*), the gene has no significant difference between the positive and negative classes when the significance level is *α*; if *D* ≥ *D*_crit_, there is a significant difference between the positive and negative samples at the 1 − *α* confidence level.

From (2), we can see that the bigger the *D* value, the greater the difference between the positive and negative classes of the gene, indicating a stronger ability to distinguish between the positive and negative samples.

### 2.3. Correlation-Based Feature Selection (CFS)

The correlation feature selection (CFS) method evaluates subsets of features according to the following hypothesis: “good feature subsets contain features that are highly correlated with the classification yet uncorrelated to each other.” The bias of the evaluation function is towards subsets containing features that are highly correlated with the class and uncorrelated with each other. Irrelevant features should be ignored because they have a low correlation with the class. Redundant features should be removed, as they will be highly correlated with one or more of the remaining features. The acceptance of a feature depends on the extent to which it predicts classes in areas of the instance space not already predicted by other features.

### 2.4. K-S Test-CFS Method for Gene Selection

As we previously mentioned, the K-S test is a general and successful attribute estimator and is able to effectively provide quality estimates of attributes in problems that have dependencies between attributes. However, the K-S test does not explicitly reduce the redundancy in selected genes. CFS selects genes that have the highest relevance with the target class and that are also maximally dissimilar to each other. Thus, the integration of the K-S test and CFS leads to an effective gene selection scheme.

The details of the K-S test-CFS algorithm are as follows: in the first stage, the K-S test is applied to find a candidate gene set. This approach removes many unimportant genes and reduces the computational load for CFS. In the second stage, the CFS method is applied to directly and explicitly reduce the redundancy and to select a compact yet effective gene subset from the candidate set.

### 2.5. Software Package

In this paper, the K-S test,* T* test, and Wilcoxon test algorithms are implemented using MATLAB R2012a. The CFS, mRMR, ReliefF, and SVM algorithms are implemented using Weka 3.6. Weka (http://www.cs.waikato.ac.nz/ml/weka/) is a software packaged that collects various types of learning algorithms for data mining tasks. The SVM algorithm uses a linear kernel function, and the penalty factor *C* takes a fixed value of 1.

## 3. Results and Discussion

### 3.1. Comparison of the K-S Test with the* T* Test and the Wilcoxon Test

This section compares the performance of the gene selection algorithms using the K-S test, the Wilcoxon test, and the* T*-test. First, the significance level alpha was set, and then, each gene in the dataset was tested by the K-S test, the Wilcoxon test, and the* T* test to select the important genes in order to form a subset of preselected genes. In the preselected gene subset, SVM was used as the classifier to calculate the accuracy of the 10-fold cross-validation. Then, a performance comparison of the gene subsets selected by the K-S test, the Wilcoxon test, and the* T* test in the different alpha values was performed.  Table 2 lists the number of gene subsets selected by the K-S test, the Wilcoxon test, and the* T* test in the five datasets with different alpha values.  Table 3 shows the average classification accuracy of the 10-fold cross-validation in the gene subsets selected by the K-S test, the Wilcoxon test, and the* T*-test in the five datasets with different alpha values.

The experimental results in Table 2 show that the number of gene subsets selected by the K-S test, the Wilcoxon test, and the* T* test with the same alpha value was different. As shown in Table 2, the K-S test selected a smaller subset of genes in most cases.

Table 2 also shows that the subset of genes selected by the three test algorithms was smaller when the confidence level was large and the significance level *α* was small. When the confidence level was 99.9%, the significance level *α* = 0.001. In the colon dataset, the size of the selected subset of genes was approximately 50, which is approximately 2.5% of the original dataset. The size of the subset of genes selected in the breast cancer dataset was approximately 1.5% of the original number of genes in the dataset. The worst case observed was with the lung cancer dataset, and at this significant level, the size of the selected gene subset for the three test algorithms was approximately 10% of the original gene number of genes in the dataset.

The above analysis shows that the K-S test is a very effective genetic importance measurement algorithm. This test selected a smaller subset of genes that had a high interclass discrimination ability.

The average classification accuracy of the subset of genes selected by the three test algorithms at the different levels of significance is shown in Table 3. For the breast cancer dataset, the significance level was 0.001, and the average classification accuracy rate of the K-S test was slightly worse than that of the Wilcoxon test; however it was better than that of the* T* test. When the significance level was 0.0.5, 0.01, or 0.005, the average classification accuracy rate of the K-S test was not lower than the rates of the Wilcoxon test and the* T* test. For the other four genetic datasets, regardless of whether the significance level was 0.05, 0.01, 0.005, or 0.001, the average classification accuracy rate of the gene subset selected by the K-S test was not lower than the rates of the Wilcoxon test and the* T* test. Therefore, this finding demonstrated that the K-S test could select a better gene subset.

Based on the above results, the K-S test was superior to the Wilcoxon test and the* T* test for gene selection.

### 3.2. Compare the CFS with the mRMR and ReliefF Algorithms

The CFS algorithm was compared to the mRMR and ReliefF algorithms to validate the performance of the gene selection in the preselected gene subset. First, all of the genes were prescreened by the K-S test with a significance level of 0.01, and a preselected gene subset was obtained. The CFS algorithm selected the appropriate subset of genes directly from the subset of prescreened genes. The mRMR and ReliefF algorithms selected the first 50 genes sorted by the importance of the gene. Then, a forward selection algorithm was used to select the appropriate subset of genes from those 50 genes.

In the experiment, we adopted SVM as a classifier and used the criteria of the average accuracy of a tenfold cross-validation in the dataset to evaluate the performance of the classifiers on the selected gene subsets. To obtain statistically significant experimental results, the dataset samples were randomly shuffled, the procedure was repeated 10 times, and the average of the 10 replicates was recorded and compared.  Table 4 shows the average accuracy of the tenfold cross-validation of the three algorithms in the five gene datasets and the corresponding number of genes on average.

From the comparison of the average accuracy (calculated from the results of the ten replicates) of the three algorithms shown in Table 4, we can see that, for the breast cancer dataset, the CFS algorithm achieves the best performance with the least features, which is significantly better than the performance of the other algorithms. For the colon dataset, the CFS was superior to the ReliefF and mRMR algorithms. For the lung cancer, ovarian, and leukemia datasets, the performance of the CFS algorithm is similar to that of the mRMR algorithm and better than that of the ReliefF algorithm.

Based on the above results, the CFS algorithm is superior to the mRMR and ReliefF algorithms for the preselected gene subset.

### 3.3. Comparison of the K-S Test-CF Algorithm with the K-S Test, CFS, mRMR, and ReliefF Algorithms

We also compared the K-S test-CFS selection algorithm with other gene selection algorithms, including the K-S test, mRMR, CFS, and ReliefF.  Table 5 presents the classification accuracy comparison using the SVM classifier based on the selected genes and these five feature selection methods. From Table 5, we observed the following:

- The K-S test-CFS algorithm achieved a better performance than the other gene selection algorithms on almost all datasets. The experimental comparisons demonstrate the effectiveness of the integration of the K-S test and CFS.
- CFS achieved a good performance on most of the datasets. However, its performance was not always as good as that of the K-S test-CFS algorithm. It outperforms the mRMR and ReliefF algorithms.

In summary, the performance of the K-S test-CFS is superior to other gene filtering algorithms. However, in the course of the experiment, we found that the runtime of the K-S test-CFS had no advantage over the other algorithms. Therefore, the focus of the next step in this work should be how to optimize the running time of the K-S test-CFS algorithm.

## 4. Conclusions

In this paper, we present a K-S test-CFS selection algorithm developed by combining the K-S test and CFS. The K-S test effectively provided quality estimates of the attributes in problems that have dependencies between attributes, and the CFS method selected genes that had the highest relevance with the target class and are also maximally dissimilar to each other. The integration of the K-S test and CFS thus leads to an effective gene selection scheme. In the first stage, the K-S test is applied to find a candidate gene set. In the second stage, CFS is applied to select a compact yet effective gene subset from the candidate set. Comprehensive experiments were conducted to compare the K-S test-CFS selection algorithm to the K-S test, CFS, ReliefF, and mRMR feature selection methods using the SVM classifier on five different datasets. The experimental results show that the K-S test-CFS gene selection is an effective method compared to the K-S test, CFS, mRMR, and ReliefF algorithms.

## Figures and Tables

**Table 1:** Dataset.

Dataset	Samples	Genes
Breast cancer	97	24481
Lung cancer	181	12533
Colon tumor	62	2000
Ovarian cancer	253	15154
Leukemia	72	7129

**Table 2:** The number of genes selected by K-S test, Wilcoxon test, and *T* test in five datasets with different alpha.

Dataset	Algorithm	Alpha = 1	Alpha = 0.05	Alpha = 0.01	Alpha = 0.005	Alpha = 0.001
Breast cancer	K-S	24481	3502	1397	940	349
	Wilcoxon	24481	3829	1529	1029	381
	*T*	24481	3251	1161	726	273
Lung cancer	K-S	12533	2886	1982	1588	1300
	Wilcoxon	12533	3225	2658	1986	1528
	*T*	12533	3190	2580	1996	1625
Colon tumor	K-S	2000	324	146	105	44
	Wilcoxon	2000	387	188	140	59
	*T*	2000	389	171	113	53
Ovarian cancer	K-S	15154	7268	3408	1386	268
	Wilcoxon	15154	7652	3927	1876	329
	*T*	15154	7900	3848	1938	318
Leukemia	K-S	7129	1716	1036	843	524
	Wilcoxon	7129	1860	1169	962	644
	*T*	7129	1811	1115	931	583

**Table 3:** The average classification accuracy (%) of 10-fold cross-validation in the gene subsets selected by K-S, Wilcoxon test, and *T*-test in five datasets with different alpha.

Dataset	Algorithm	Alpha = 1	Alpha = 0.05	Alpha = 0.01	Alpha = 0.005	Alpha = 0.001
Breast cancer	K-S	68.6	83.6	86.3	86.3	83.2
	Wilcoxon	67.8	83.5	84.8	84.8	84.8
	*T*	66.7	80.2	83.5	80.5	80.5
Lung cancer	K-S	85.8	89.6	90.4	91.6	91.6
	Wilcoxon	85.8	86.9	88.5	89.5	89.5
	*T*	83.6	86.9	88.5	89.5	89.5
Colon tumor	K-S	73.4	81.4	85.9	85.9	83.2
	Wilcoxon	73.4	79.2	80.2	81.5	83.2
	*T*	73.4	79.2	80.2	81.5	81.5
Ovarian cancer	K-S	95.3	98.6	100	100	96.5
	Wilcoxon	95.3	97.3	98.6	100	94.6
	*T*	95.3	97.3	98.6	100	94.6
Leukemia	K-S	71.6	75.3	81.4	82.6	85.6
	Wilcoxon	71.6	75.3	81.4	81.4	83.5
	*T*	71.6	75.3	81.4	81.4	82.2

**Table 4:** The comparisons in CFS, mRMR, and ReliefF algorithms.

Dataset	Gene selection method					
	CFS		mRMR		ReliefF	
	The number of genes	Accuracy	The number of genes	Accuracy	The number of genes	Accuracy
Breast cancer	11.7	87.4	10.4	85.6	15.9	59.5
Lung cancer	23.2	91.6	25.7	88.4	26.7	87.6
Colon tumor	10.7	90.1	12.6	86.4	15.3	84.8
Ovarian cancer	33.2	98.5	31.5	95.6	37.4	93.2
Leukemia	25.2	99.6	2.5	99.6	16.4	77.6

**Table 5:** The comparisons in K-S test-CF, K-S, CFS, mRMR, and ReliefF algorithms.

Dataset	Gene selection method									
	k-S test- CFS		CFS		k-S test		mRMR		ReliefF	
	The number of genes	Accuracy	The number of genes	Accuracy	The number of genes	Accuracy	The number of genes	Accuracy	The number of genes	Accuracy
Breast cancer	11.7	87.4	19.6	80.5	22.5	78.8	21.8	82.4	15.9	59.4
Lung cancer	23	91.6	27.3	88.9	33.4	80.6	289	89.8	33.6	84.7
Colon tumor	10.7	90.1	6.8	89.7	19.4	84.5	5.9	89.7	15	74.9
Ovarian cancer	33.2	98.5	31.6	95.3	46	78.9	32.7	95.2	39.6	90.6
Leukemia	25.2	79.6	33.3	78.9	38.7	72.7	28.6	75.7	36.4	77.6
